# Next-Generation Nafion Membranes: Synergistic Enhancement of Electrochemical Performance and Thermomechanical Stability with Sulfonated Siliceous Layered Material (sSLM)

**DOI:** 10.3390/polym17131866

**Published:** 2025-07-03

**Authors:** Valeria Loise, Cataldo Simari

**Affiliations:** Department of Chemistry and Chemical Technologies, University of Calabria, 87036 Rende, Italy; valeria.loise@unical.it

**Keywords:** Nafion, nanocomposite membranes, sulfonated silica layers, PFG NMR, proton conductivity, H_2_/O_2_ fuel cells

## Abstract

Nafion, while a benchmark proton exchange membrane (PEM) for fuel cells, suffers from significant performance degradation at elevated temperatures and low humidity due to dehydration and diminished mechanical stability. To address these limitations, this study investigated the development and characterization of Nafion nanocomposite membranes incorporating sulfonated silica layered materials (sSLMs). The inherent lamellar structure, high surface area, and abundant sulfonic acid functionalities of sSLMs were leveraged to synergistically enhance membrane properties. Our results demonstrate that sSLM incorporation significantly improved ion exchange capacity, water uptake, and dimensional stability, leading to superior water retention and self-diffusion at higher temperatures. Critically, the nanocomposite membranes exhibited remarkably enhanced proton conductivity, particularly under demanding conditions of 120 C and low relative humidity (i.e., 20% RH), where filler-free Nafion largely ceases to conduct. Single H_2_/O_2_ fuel cell tests confirmed these enhancements, with the optimal sSLM-Nafion nanocomposite membrane (N-sSLM_5_) achieving a two-fold power density improvement over pristine Nafion at 120 C and 20% RH (340 mW cm^−2^ vs. 117 mW cm^−2^ for Nafion). These findings underscore the immense potential of sSLM as a functional filler for fabricating robust and high-performance PEMs, paving the way for the next generation of fuel cells capable of operating efficiently under more challenging environmental conditions.

## 1. Introduction

Nafion, a perfluorosulfonic acid (PFSA) polymer, is unequivocally recognized as the gold standard material for proton exchange membranes (PEMs) in low-temperature fuel cells [1,2]. Its remarkable attributes stem from its unique chemical architecture, consisting of a hydrophobic perfluorinated backbone and hydrophilic sulfonic acid side chains. This distinct molecular design leads to a nanoscale phase separation, forming an intricate network of interconnected ionic clusters and channels [3]. Within these hydrophilic domains, water molecules are absorbed, facilitating efficient proton transport primarily via two mechanisms: the Grotthuss mechanism (proton hopping through a hydrogen-bonded water network) and the vehicular mechanism (proton diffusion with water molecules) [4,5]. These transport pathways, coupled with Nafion’s inherent robust chemical stability against radical attack and its good mechanical strength under hydrated conditions, have cemented its position as the preferred membrane material for many years [6,7].

Despite its advantages, the widespread commercialization of Nafion-based PEM fuel cells for various applications, especially those operating at higher temperatures (>80 °C) and reduced relative humidity (RH), faces significant hurdles due to its intrinsic limitations [8,9]. At elevated temperatures, the vapor pressure of water increases, leading to severe dehydration of the membrane. This desiccation causes a drastic reduction in the ionic conductivity as the continuous water network essential for proton transport is disrupted [9]. Furthermore, high temperatures accelerate polymer chain mobility, which can result in increased gas crossover (e.g., H_2_ and O_2_), leading to fuel efficiency losses and safety concerns. Concurrently, issues such as excessive membrane swelling under fully humidified conditions and a pronounced decrease in mechanical integrity and durability at higher temperatures contribute to substantial challenges, including delamination, cracking, and ultimately, a shortened operational lifespan of the fuel cell stack [10,11]. These drawbacks necessitate complex and energy-intensive external humidification systems and limit the overall power density and thermal efficiency of the fuel cell system.

To circumvent these formidable challenges and broaden the operational envelope of PEM fuel cells, extensive research efforts have been dedicated to engineering Nafion-based nanocomposite membranes [2,12]. This strategy involves the strategic incorporation of various inorganic or organic nanofillers into the Nafion matrix, aiming for a synergistic amalgamation of properties. The rationale is to leverage the unique functionalities of the fillers to directly mitigate Nafion’s deficiencies while preserving its core advantages. Common approaches in the literature include the following: (i) the incorporation of inorganic proton conductors such as phosphotungstic acid (PWA) [13,14], zeolites [15,16,17], or metal–organic frameworks (MOFs) [18,19,20], which provide additional acidic sites and pathways for proton transport, even under dehydrated conditions; (ii) the dispersion of hygroscopic nanoparticles like silicon dioxide (SiO_2_) [21,22], titanium dioxide (TiO_2_) [23,24], or various clay minerals [25,26,27,28], which improve water retention capabilities by increasing the number of water-binding sites within the membrane, thereby maintaining higher hydration levels at elevated temperatures and low RH [29]; (iii) the use of materials capable of reducing gas crossover, such as graphene oxide (GO) [30,31], carbon nanotubes [32], and smectite clay [33], whose tortuous pathways can impede gas diffusion. These fillers are typically chosen for their ability to reinforce the polymer matrix, enhance dimensional stability by restricting polymer chain movements, and provide additional ion exchange sites or water-trapping capacities, thereby improving overall membrane performance and durability under demanding operating conditions [34]. However, among them, many present limitations. For instance, some fillers offer improved mechanical strength but may compromise proton conductivity, especially under low-humidity conditions due to their limited proton-conducting pathways or poor interface compatibility with the Nafion matrix. Others might provide additional water retention but suffer from issues like aggregation at higher loadings or insufficient chemical stability in a harsh fuel cell environment. The challenge lies in identifying a filler that can simultaneously contribute to both electrochemical performance (particularly proton conductivity at elevated temperatures and reduced humidity) and thermomechanical stability without introducing detrimental side effects like increased membrane swelling or poor dispersion.

It is precisely to address this multi-faceted challenge that our investigation specifically focuses on the integration of sulfonated silica layered materials (sSLMs) as highly promising functional fillers within the Nafion matrix. The fundamental structural, morphological, and physico-chemical characteristics of sSLMs, including their lamellar structure, high surface area, and the presence of a high density of sulfonic acid functionalities, have been comprehensively detailed and validated in our previously published work [35]. Unlike many conventional fillers, sSLM uniquely combines several critical attributes. It possesses an inherent layered structure that can facilitate the transport of protons along its interlayer spaces, thereby offering intrinsic proton conductivity in addition to providing sites for water retention. The successful sulfonation of these materials ensures the presence of sulfonic acid functional groups, which are critical for establishing continuous proton pathways and enhancing the ion exchange capacity of the composite membrane. Furthermore, the siliceous nature of sSLM contributes to improved thermomechanical stability, acting as a robust reinforcing agent within the polymer matrix. Crucially, the unique combination of a layered structure with tailored sulfonation provides a synergistic effect: the layered morphology can potentially guide and enhance proton transport within the membrane, while the sulfonic groups ensure a high density of charge carriers. This dual functionality is often not as pronounced or optimized in single-component fillers or those with less-defined internal structures, making sSLM a particularly promising candidate for next-generation Nafion membranes operating under demanding conditions. Leveraging these inherent advantages, particularly the sSLM’s capacity for strong interactions with water and the Nafion polymer, the present study aims to rigorously investigate the profound impact of incorporating sSLMs into Nafion membranes. We seek to demonstrate the synergistic enhancement in proton conduction, refined water management, improved dimensional stability, and superior thermomechanical robustness afforded by sSLM, especially under the critical high-temperature and low-humidity operating conditions relevant for next-generation fuel cells. Through a thorough and multi-faceted evaluation of key parameters, including ion exchange capacity (IEC), water uptake (WU), water self-diffusion coefficients measured by PFG NMR, proton conductivity under various environmental conditions (temperature and relative humidity), and dynamic mechanical analysis (DMA), followed by a conclusive validation through single H_2_/O_2_ fuel cell performance tests under both mild and aggressive operating conditions, we delineate the significant potential of Nafion–sSLM nanocomposite membranes as promising candidates for high-performance fuel cell applications.

## 2. Materials and Methods

### 2.1. Materials

Nafion (20 wt% dispersion in water and lower aliphatic alcohols), N,N-Dimethylacetamide (DMAc), and NaOH (0.01 M, volumetric standard) were purchased from Sigma-Aldrich, Milan, Italy. 3-(trihydroxysilyl)propyl-1-propane-sulfonic acid (30–35% in water) was purchased from Gelest (Morrisville, PA, USA).

### 2.2. Synthesis of Sulfonated Siliceous Layered Materials (sSLMs)

The detailed synthesis of the organosilica layered material, along with comprehensive characterization data, including X-ray Diffraction (XRD) to confirm its layered structure and Fourier transform infrared (FTIR) spectroscopy to verify the successful introduction of sulfonic acid functional groups, has been thoroughly reported in our prior works [36,37]. Briefly, an aqueous solution of 3-(trihydroxysilyl)propyl-1-propane-sulfonic acid was placed in a Teflon beaker and allowed to dry, resulting in a transparent, cracked xerogel monolith. Deionized water was then introduced to the xerogel, forming a milky suspension. This suspension was subjected to centrifugation at 9000 rpm for 10 min. The isolated gel was subsequently washed five times with water and two times with acetone to obtain a fine, white powder, which was labeled as sSLM.

### 2.3. Preparation of Nanocomposite Membrane

**Scheme 1:**
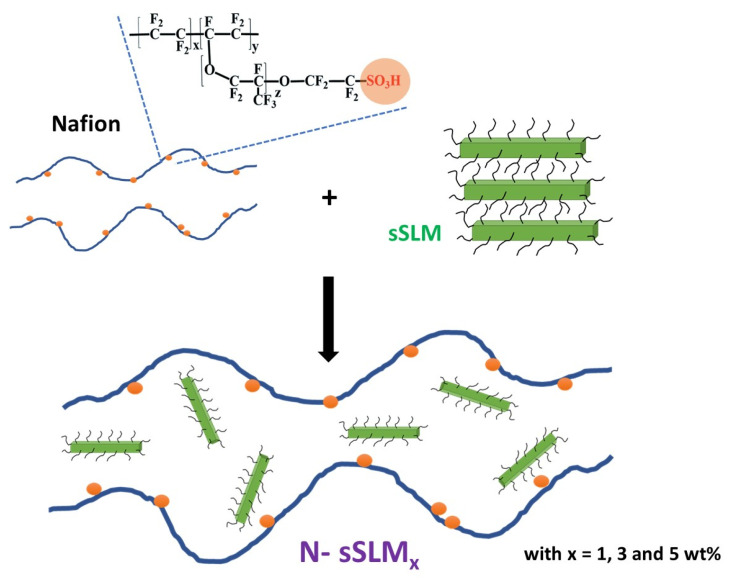
Representation of the preparation procedure for N-sSLM membranes.

Composite membranes were fabricated using a 20 wt% Nafion solution, which was heated to 60 °C to evaporate the solvents, then re-dissolved in 10 mL of dimethylformamide (DMF) to yield a clear solution. Separately, the appropriate amount of sSLM, from 0 to 5 wt% with respect to the polymer, was dispersed in 2 mL of DMF for 24 h by alternating stirring and sonication. Thereafter, the fine dispersion was added dropwise to the Nafion solution. This mixture was continuously stirred at 60 °C to ensure thorough blending (see Scheme 1). The resulting solution was then cast onto a Petri dish and dried overnight at 100 °C to form a homogenous membrane with a thickness of approximately 50 μm. Both the filler-free Nafion membrane (prepared via the same casting method) and the composite membranes underwent a standard thermal and acid activation process [38,39]. N-sSLM at higher filler loadings, i.e., 8–10 wt%, were also prepared but filler agglomeration made them non-homogeneous and too brittle to be tested.

### 2.4. Characterization Techniques

The ion exchange capacity (IEC) for membranes, incorporating filler loadings from 0% to 3%, was established through an acid–base titration procedure [3]. Initially, acid-activated membrane samples underwent a 24 h immersion in a 2 M NaCl solution at room temperature. This crucial step enabled the complete exchange of H^+^ ions for Na^+^ ions. Subsequently, the released H^+^ ions were volumetrically titrated against a standardized 0.01 M NaOH solution, with phenolphthalein serving as the endpoint indicator. The IEC results, reported in milliequivalents per gram (meq/g), were derived from Equation (1):

(1) $$\mathrm{IEC}\;(\mathrm{meq}/g)=\frac{M_{NaOH}\;V_{NaOH}}{W_{dry}}$$

In this equation, W_dry_ denotes the dry mass of the membrane, while M and V represent the molar concentration (mol/L) and volume (in mL) of the NaOH solution expended during titration to neutralize the H^+^ ions.

Water uptake (WU%) measurements commenced with the preparation of dry membrane samples (W_dry_). These dried membranes were then submerged in distilled water for 24 h at room temperature to facilitate complete saturation. Following the soaking period, any residual surface water was carefully blotted away with filter paper, and the hydrated membranes were immediately weighed (W_wet_). The percentage of water uptake was then calculated by Equation (2):

(2) $$\mathrm{WU}\%=\frac{Wwet-Wdry}{Wdry}*100$$

Furthermore, the influence of temperature on water uptake was examined by equilibrating membranes in distilled water heated to a series of temperatures ranging from 30 to 80 °C, with 10 °C increments. A 2 h equilibration time was maintained at each temperature before measurements were taken.

The lambda value (λ), which estimates the number of H_2_O molecules per -SO_3_H group [40], is calculated according to Equation (3):

(3) $$\lambda =\frac{wu}{IEC*{Mw}_{H_{2}O}\;}$$

For DMA, a Metravib DMA/25 system (Paris, France), equipped with a shear jaw specifically designed for films, was utilized. The measurements were conducted across a temperature range of 25–200 °C. A dynamic stress of amplitude 10^−3^ was applied at a frequency of 1 Hz, with the temperature increasing at a steady rate of 2 °C/min.

A Bruker Avance 300 Spectrometer (Billerica, MA, USA), fitted with a 30 G/cm/A multinuclear probe and exchangeable RF inserts, was used for NMR characterization. Pulsed Field Gradient-stimulated echo sequence (PFG-STE) was used to measure the self-diffusion coefficients (D) of water filling the hydrophilic domains of the PEMs. The sequence involves three 90° RF pulses (π/2 − τ_1_ − π/2 − τ_m_ − π/2) and two gradient pulses applied after the first and third RF pulses. The echo is detected at time τ = 2τ1 + τm. Echo amplitude attenuation was analyzed using the Stejskal–Tanner equation (Equation (4)) [41]:

(4) $$I=I_{0}\;e^{-[{\left(\gamma g\delta \right)}^{2}\;\left(\Delta -\frac{\delta }{\;3}\right)D]}$$

Here, I and I_0_ represent the signal intensity with and without a gradient, respectively. D is the diffusion coefficient, γ is the gyromagnetic ratio, g is the field gradient, δ is the gradient pulse duration, and Δ is the time delay. Experimental parameters included δ = 0.75 ms and Δ = 7.5 ms. The gradient amplitude was increased in 12 steps from 50 to 8000 G/cm. The procedure for sample preparation was already described elsewhere [42]. All D values were collected by increasing the temperature from 20 to 130 °C in 20 °C increments, allowing 15 min of sample equilibration at each step.

The proton conductivity of all membranes was measured though Electrochemical Impedance Spectroscopy (EIS). Tests took place at 120 °C across varying relative humidities (RHs), from 20% to 100% and at 90% RH with temperature in the range 20–120 °C. For these measurements, we used a custom-built, two-electrode cell connected to an 850 C fuel cell test hardware (Scribner Associates, Inc., Southern Pines, NC, USA) in the case of through-plane configuration. In-plane conductivity was measured using a BT-115 4-electrodes Conductivity Cell. A PGSTAT 30 potentiostat/galvanostat (Methrom Autolab, Utrecht, The Netherlands), equipped with an FRA module, measured the cell’s AC impedance response. The AC voltage amplitude was set at 10 mV, and the frequency ranged from 1 Hz to 1 MHz. NOVA software 2.0 was used to determine the membrane resistance (R) from the high-frequency intersection of the impedance arc with the real axis on the Nyquist plot. Proton conductivity, σ (S/cm), was then calculated with Equation (5):

(5) $$\sigma =\frac{d}{R*A\;}$$

Here, A represents the area (calculated as membrane thickness multiplied by height), and d (cm) is the distance between the two electrodes.

Electrodes were manufactured using the casting knife method [43]. The catalytic ink was prepared by mixing commercial platinum catalyst (Pt/C, 40% carbon-supported, Alfa Aesar, Hefril, MA, USA) with 33% Nafion ionomer (20% solution, Ion Power, Hampshire, UK). Ultrasounds were used to ensure a good dispersion of the ink. Afterward, the ink was spread onto the backing layer of a Sigracet 25-BC Gas Diffusion Layer (SGL). Both the anode and cathode had a platinum loading of 0.5 mg cm^−2^. Membrane Electrode Assemblies (MEAs) were formed by pressing the prepared electrodes onto each membrane type. This was accomplished at a pressure of 30 kg cm^−2^ for 2 min at 130 °C.

For single H_2_/O_2_ fuel cell polarization measurements, galvanostatic tests were carried out under steady-state conditions using a custom-built test bench equipped with an electronic load (Fideris, Vancouver, WA, USA, 125 W, 20 V, 5 A). The MEAs were tested in a 5 cm^−2^ single-cell configuration. Temperatures ranged from 80 °C to 120 °C, and relative humidity (RH) varied between 100% and 25%. H_2_ and O_2_ were supplied to the anode and cathode, respectively, at atmospheric pressure. The flow rates for H_2_ and O_2_ were set at 1.5 and 2.0 stoichiometry, respectively, based on a current density of 2.00 A cm^−2^. These flow rates were kept constant throughout the experiments using mass flow controllers (Brooks Instruments, Hatfield, PA, USA). Specific sensors allowed for precise control of the cell’s temperature and humidity.

## 3. Results and Discussion

**Figure 1:**
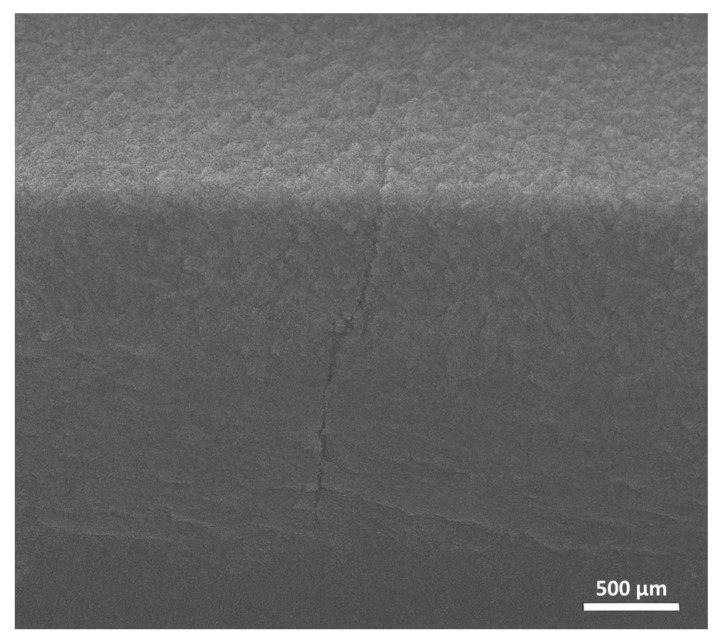
FE SEM image of the cross-section for the cryo-fractured N-sSLM_5_ membrane.

Figure 1 illustrates the cross-sectional FE-SEM image for the N-sSLM_5_, revealing a homogeneous and dense microstructure. Critically, no significant filler accumulation or large-scale phase separation is observed, indicating good interface compatibility between sSLM and the Nafion matrix. Examination of the cleavage plane, which appears relatively smooth and uniform, indirectly suggests either excellent sSLM dispersion at sub-micrometer scales with a minor degree of preferential orientation of the lamellae, possibly along the in-plane direction during fabrication. Such an alignment could contribute to the membrane’s anisotropic properties and enhance in-plane proton transport.

**Figure 2:**
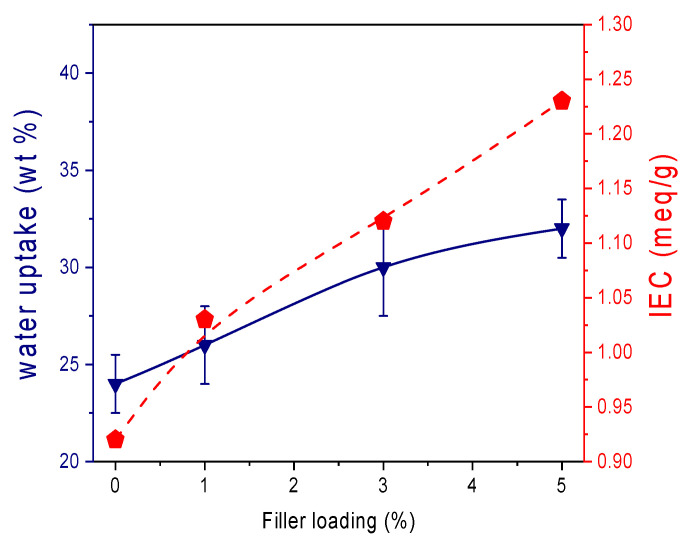
Variations in filler loading of IEC and water uptake for sSLM nanocomposite.

For polymer electrolyte membranes (PEMs) in fuel cells, both ion exchange capacity (IEC) and water uptake (WU) are critical parameters. IEC, which represents the concentration of fixed charge sites, directly relates to the number of available sites for proton conduction. Adequate water uptake is essential for proton transport, as water acts as the primary carrier for protons via mechanisms such as the Grotthuss and vehicular mechanisms. Optimizing these properties is key to achieving high proton conductivity and overall fuel cell performance. Figure 2 illustrates the significant impact of filler loading on the IEC and water uptake of the sSLM material, offering insights into how composite membranes can be engineered for enhanced performance. As observed, both water uptake (blue, left y-axis) and IEC (red, right y-axis) exhibit a clear increasing trend with increasing filler loading, indicating that the incorporation of the filler effectively enhances the membrane’s ability to exchange ions and thus its hydrophilicity. Specifically, the IEC rises proportionally from about 0.94 meq/g at 0% filler loading to approximately 1.23 meq/g at 5% filler loading due to the presence of a large number of sulfonic acid functionalities on the sSLM surface, which introduces additional hydrophilic sites. Concurrently, the water uptake increases from approximately 24 wt% at 0% filler loading to around 32 wt% at 5% filler loading. This synergy implies that the incorporated filler successfully introduces additional sites for water absorption and ion exchange, or it favorably alters the polymer matrix in a way that facilitates these processes, ultimately contributing to improved proton conductivity in the membrane. The outcome is a promising indicator for the development of high-performance PEMs.

**Figure 3:**
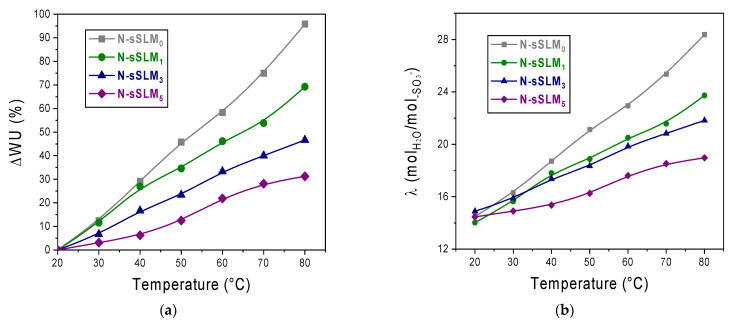
Temperature variations in (**a**) normalized WU variation (ΔWU) and (**b**) lambda (mol_H2O_/mol_SO3H_) for Nafion-based membranes.

Figure 2 provides crucial insights into the thermo-hydrophilic stability of the Nafion-based membranes, directly complementing the data of IEC and WU. The temperature-dependent behavior of water uptake (ΔWU) and of λ, defined as the number of water molecules per sulfonate group, is illustrated in Figure 3a and Figure 3b, respectively. Consistent across all samples, both ΔWU and λ increase with rising temperature, which is typical for polymeric membranes. Thermal energy favors polymer chain segmental motion, leading to greater free volume and improved accessibility for water molecules to interact with the hydrophilic sulfonate groups. A clear and inverse relationship is observed between the filler loading and membrane properties. Pristine Nafion (N-sSLM_0_) consistently exhibits the highest WU variation and λ values across the entire temperature range. Yet, dimensional stability increases with filler loading and N-sSLM_5_ shows the lowest water uptake variation and λ values. This counter-intuitive observation, especially when considering the sulfonated nature of the filler and the increased hydrophilicity of nanocomposite membrane at RT, can be explained by several factors. The layered structure of sSLM may promote a more densely packed polymer matrix. This restricts the mobility of polymer chains and reduces the available free volume for water penetration, effectively limiting the swelling of the membrane during heating. Furthermore, the progressive reduction in total λ with increased filler loading suggests that the sSLM filler might fundamentally alter the water distribution, facilitating a higher proportion of the absorbed water being in a more stable, “bound” state. This bound water is less volatile and does not evaporate readily at elevated temperatures (e.g., 120 °C), thereby aiding in membrane hydration under demanding conditions. Moreover, such reduced sensitivity of WU to temperature fluctuations directly translates to enhanced dimensional stability across the operating temperature range. Such stability is paramount for the long-term integrity of fuel cell stacks, as it mitigates mechanical stress from hydration/dehydration cycles, a common cause of delamination and fatigue in traditional Nafion membranes. Thus, the sSLM’s presence significantly contributes to a more robust and durable MEA, marking a notable advancement despite the higher absolute water uptake.

**Figure 4:**
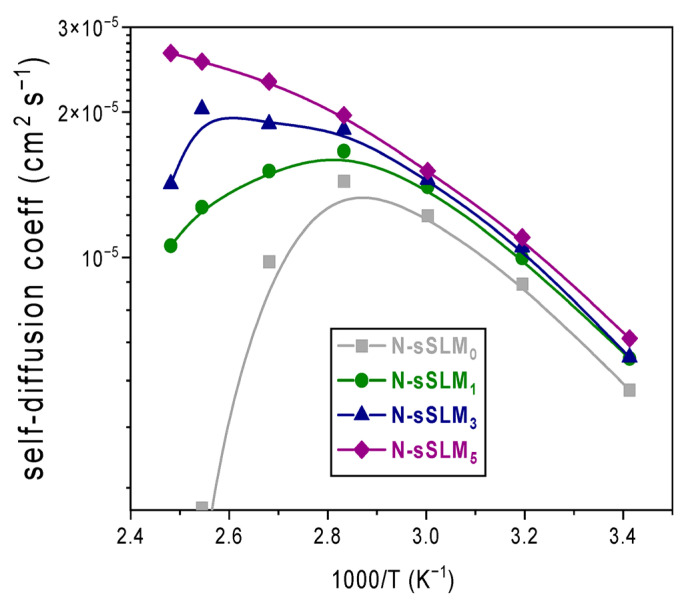
Water self-diffusion coefficient vs. temperature for N-sSLMx membranes.

The direct measurement of water self-diffusion coefficient (D) was achieved by PFG NMR technique [44]. This coefficient directly relates to proton and water mobility within the membrane and thus directly correlates with proton conductivity, a critical parameter for PEM fuel cells. Figure 4 illustrates the temperature dependence of D in the range 20–130 °C for water-saturated membranes at different filler loadings. In the range 20–80 °C, diffusivity slightly increases with filler loading, suggesting the sSLM filler might create more continuous or less tortuous proton-conducting pathways, possibly through the formation of interconnected clusters of bound water around the filler particles. Furthermore, the sSLM filler, as a layered material, might contribute significantly to surface conduction by providing a large surface area for proton hopping.

A general increase in the self-diffusion coefficient with rising temperature is observed across all samples, consistent with thermally activated molecular motion. However, the non-linear nature of these curves suggests complex diffusion mechanisms that evolve with temperature. The Nafion recast membrane exhibits an abrupt drop in D values above 80 °C due to massive evaporation of bulk-like water. Contrariwise, onset of partial dehydration is shifted toward higher temperatures in the case of nanocomposite PEMs. Notably, N-sSLM_5_ consistently displays the highest self-diffusion coefficients across the entire temperature range and maintains a continuous increase during heating. The outcome is a direct consequence of the filler’s ability to not only increase the overall hydration capacity but, more importantly, to modify the water’s state towards a more thermally stable, “bound” configuration. This bound water, while potentially limiting excessive swelling, effectively maintains the vital proton transport network at elevated temperatures, thereby offering a significant advantage for high-temperature PEM fuel cell applications.

**Figure 5:**
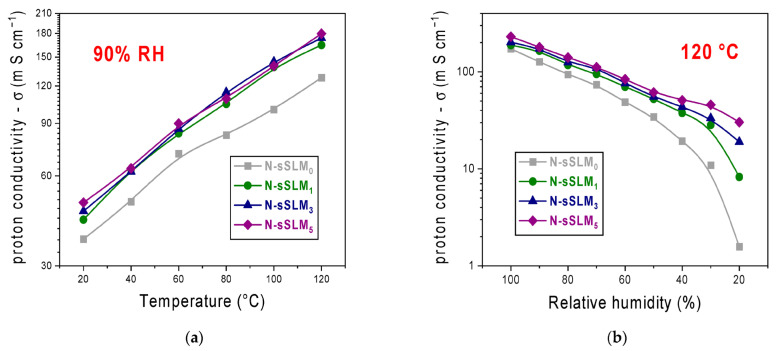
Proton conductivity for sSLM membranes (**a**) vs. temperature at 90% RH and (**b**) vs. relative humidity at 120 °C.

Measuring the proton conductivity of the various Nafion nanocomposite membranes under different environmental conditions is crucial for evaluating the potential of these PEMs in high-temperature, low-humidity PEM fuel cell applications. Figure 5a displays the proton conductivity (σ) as a function of temperature (ranging from 20 °C to 120 °C) at a constant relative humidity of 90%. All membranes exhibit an increase in conductivity with increasing temperature, which is typical due to enhanced proton mobility and water diffusion at higher thermal energy. Crucially, the conductivity of filler-free Nafion varies from 36.89 mS cm^−1^ at 20 °C to 127.91 mS cm^−1^ at 120, but the introduction of sSLM filler significantly enhances its performance even at very low filler content, i.e., 1 wt%. In this regard, N-sSLM_5_ consistently exhibits the highest conductivity across the entire temperature range, yielding from 48.89 mS cm^−1^ to179.59 mS cm^−1^ in the range 20–120 °C under full humidification. Figure 5b is even more insightful, illustrating the proton conductivity as a function of relative humidity (RH) at a high operating temperature of 120 °C. This condition is highly demanding for conventional PEMs, as water management becomes challenging. As expected, proton conductivity for all membranes decreases drastically with decreasing RH due to dehydration. However, the superior performance of the sSLM-filled membranes under these harsh conditions is striking. While the N-sSLM_0_ membrane experiences a precipitous drop in conductivity at lower RH levels (e.g., below 40%), the sSLM-filled membranes maintain remarkably higher conductivities. N-sSLM_5_ again stands out, exhibiting the highest proton conductivity even at very low RH (i.e., 30.24 mS cm^−1^ at 20%), where the filler-free Nafion mostly ceases to conduct. This observation strongly supports the earlier hypothesis that the sSLM filler promotes the formation of stable, “bound” water. This stable, bound water, in conjunction with the inherent proton-conducting properties of the sulfonated layered materials, enables the formation and maintenance of additional efficient proton pathways even under dehydrating conditions. The ability of N-sSLM_5_ to sustain high proton conductivity at 120 °C and low RH is a significant advantage for practical fuel cell applications, as it allows for simplified water management systems and improved thermal efficiency by operating above the boiling point of water. These results strongly support the strategic incorporation of sSLM fillers for developing next-generation, high-performance PEMs suitable for demanding operating environments.

**Figure 6:**
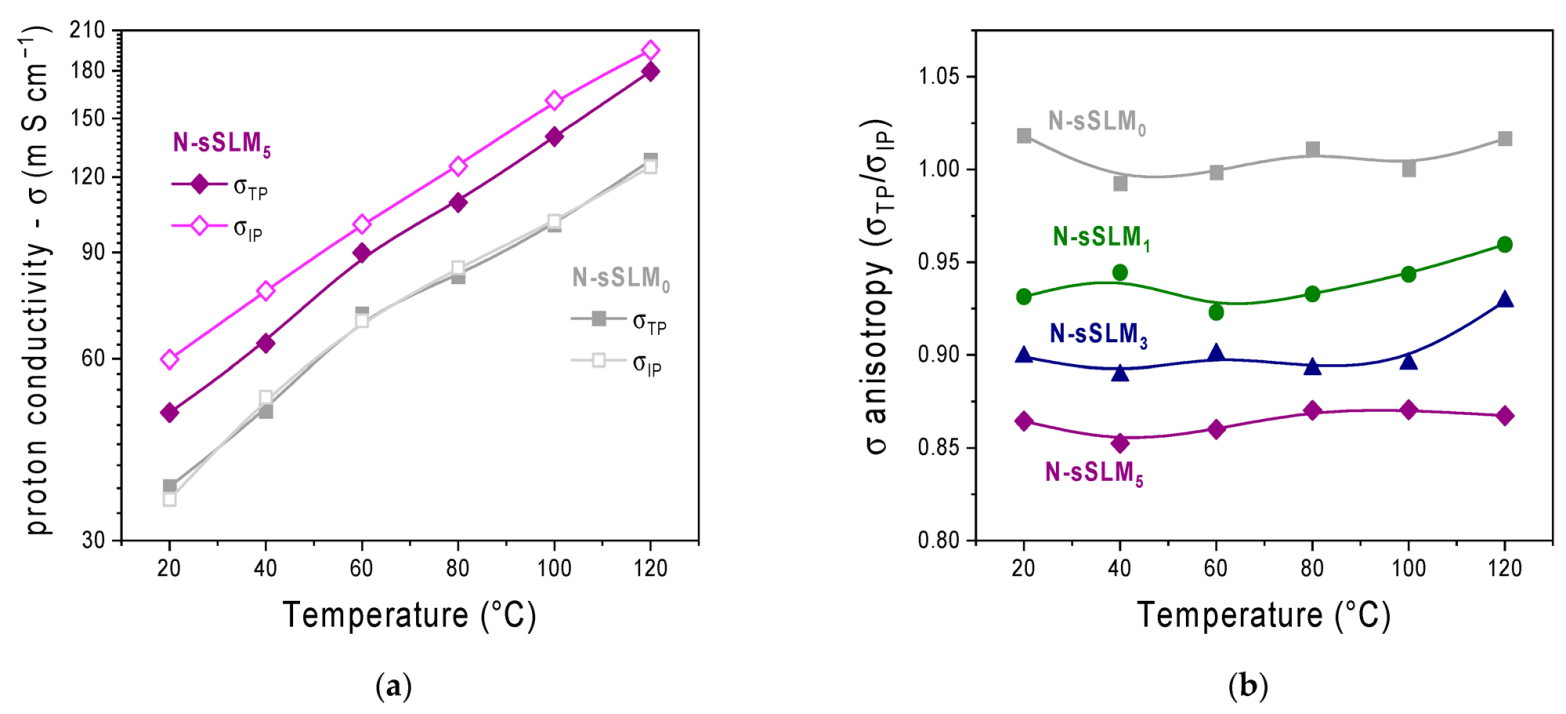
(**a**) Comparison of the in-plane (IP) and through-plane (TP) conductivities measured on filler-free Nafion and N-sSLM_5_ nanocomposites. (**b**) Anisotropy degree defined as σTP/σIP for the Nafion-based membranes.

The integration of layered materials into polymer matrices to form nanocomposite membranes often introduces conductivity anisotropy [45,46]. This phenomenon arises primarily from the preferential orientation of the 2D filler particles within the polymer during membrane processing. Consequently, proton transport pathways become more aligned along specific directions (typically in-plane) compared to others (through-plane). While such fillers can significantly enhance overall conductivity by providing additional conduction routes, this directional preference can lead to disparities between in-plane and through-plane conductivities, impacting the uniform current distribution and overall performance in fuel cell applications. Consequently, the influence of sulfonated silica layered materials (sSLMs) on the anisotropy of proton conductivity in Nafion-based membranes was rigorously investigated. In this regard, Figure 6a compares the temperature-dependent in-plane (σIP) and through-plane (σIP) proton conductivities for two representative membranes: the filler-free N-s SLM_0_ and the nanocomposite N-sSLM_5_. For N-sSLM_0_, it is evident that σIP and σTP are practically comparable across the entire temperature range, indicating the filler-free Nafion membrane possesses highly isotropic proton conductivity. In contrast, for N-sSLM_5_, a noticeable difference between σIP and σTP emerges, with σIP being consistently higher. Consequently, while the filler boosts both conduction directions, it might disproportionately enhance in-plane transport. This suggests the sSLM lamellae take preferential alignment in parallel to membrane surface during casting. Figure 6b quantifies this anisotropy by plotting the ratio of through-plane to in-plane conductivity (σTP/σIP) as a function of temperature for all investigated membranes. Clearly, a ratio closer to 1 signifies more isotropic conduction. Filler-free Nafion maintains a ratio close to 1, suggesting relatively isotropic conduction, but the anisotropy generally increases with the filler loading. This preferential alignment can lead to a trade-off where the overall proton conductivity is improved, but at the expense of isotropic transport. While increased through-plane proton conductivity is beneficial for fuel cell operation, the observed in-plane anisotropy in our modified membranes necessitates careful consideration in MEA design. Significant in-plane anisotropy can compromise the uniformity of reactant distribution across the membrane, potentially leading to non-uniform current density and localized performance issues. It may also indirectly affect vertical mass transfer and water management within the catalyst layers. Therefore, optimizing MEA design for these sSLM-modified Nafion membranes will require strategies to mitigate potential negative impacts of in-plane anisotropy, ensuring uniform operation and maximizing the benefits of enhanced through-plane proton transport.

**Figure 7:**
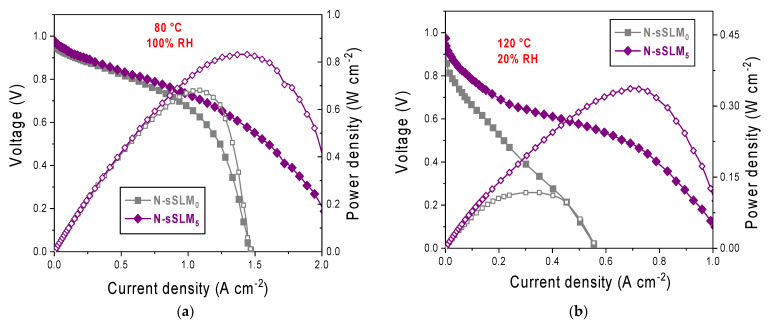
Polarization (filled symbols) and power density (empty symbols) curves under (**a**) 80 °C/100% RH and (**b**) 120 °C/20% RH.

The N-sSLM_5_, which showed the highest potential for practical application was assembled in a MEA and tested in a single H_2_/O_2_ fuel cell. For comparison, a MEA was also assembled with the N-sSLM_0_ (filler-free) benchmark and tested under the same conditions. Figure 7a illustrates the fuel cell performance under relatively mild conditions (80 °C and 100% RH), where water management is generally less challenging. Both membranes exhibit typical polarization (Voltage vs. Current Density) and power density (Power Density vs. Current Density) curves. In the activation loss region (low current density), the membranes exhibit comparable OCV but slightly higher for N-sSLM_5_, indicating the nanocomposite membrane ensures lower fuel permeability. However, as the current density increases, a clear performance divergence emerges. The N-sSLM_5_ yields a clearly higher power density of approximately 0.85 W cm^−2^ at about 1.5 A cm^−2^, whereas N-sSLM_0_ reaches a peak power density of roughly 0.68 W cm^−2^ at a similar current density before rapidly declining. The higher voltage output across the entire operating range for the nanocomposite indicates lower ohmic and mass transport resistances. The superior performance of N-sSLM_5_ is even more pronounced under more challenging conditions, i.e., 120 °C, 20% RH (Figure 7b). These conditions simulate real-world high-temperature, low-humidity operation and severely test the membrane’s ability to retain water and maintain proton conductivity. Under these demanding circumstances, the filler-free Nafion experiences a drastic reduction in performance: its peak power density is severely limited to approximately 115 mW cm^−2^ at a very low current density before mass transport limitations and dehydration lead to a rapid voltage drop. This aligns perfectly with the sharp decline in proton conductivity observed for N-sSLM_0_ at low RH. In stark contrast, the N-sSLM_5_ membrane demonstrates remarkable resilience. It achieves a peak power density of approximately 340 mW cm^−2^ at about 0.8 A cm^−2^, maintaining a much higher voltage output across the entire current density range. This represents a 2-fold performance improvement and a superior performance compared to the benchmark. The sSLM filler’s ability to promote and retain thermally stable “bound” water directly translates into superior fuel cell performance. This robust performance under challenging conditions validates the potential of these sSLM–Nafion nanocomposite membranes as promising candidates for next-generation PEM fuel cells, enabling higher operating temperatures and simplified humidification systems, thereby improving overall system efficiency and reducing the overall device cost.

**Figure 8:**
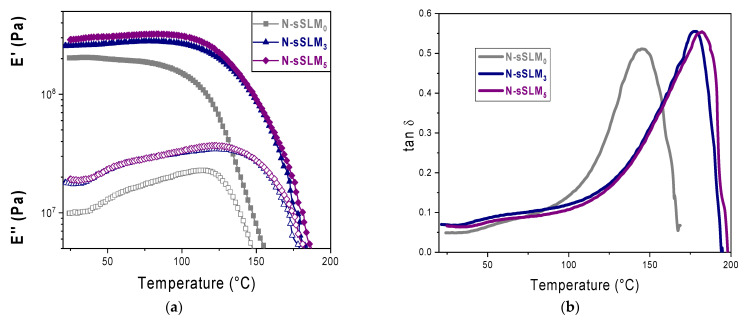
Temperature evolution of the (**a**) storage (filled symbols) and loss (empty symbols) moduli and (**b**) dumping factor (tan δ) in the range of 20–200 °C.

Assessing the viscoelastic properties is crucial for understanding the mechanical stability, flexibility, and glass transition behavior of the membranes, particularly regarding their durability and performance in fuel cell applications. Figure 8 presents the dynamic mechanical analysis (DMA) results for the N-s SLM membranes, specifically showing the temperature dependence of the storage modulus (E′), loss modulus (E″), and damping factor (tan δ). Figure 8a displays both the storage modulus (E′) and loss modulus (E″) as a function of temperature. While storage modulus relates to the elastic component of the material, reflecting its stiffness, the loss modulus represents the viscous component, reflecting the energy dissipated as heat during deformation. For all samples, E’ generally decreases with increasing temperature, indicating a reduction in stiffness as the material softens. However, the incorporation of sSLM filler significantly enhances the storage modulus, and N-sSLM_5_ consistently exhibits the highest E′ values across the entire temperature range. This indicates that the sSLM filler acts as a reinforcing agent, improving the mechanical rigidity and dimensional stability of the Nafion matrix. With regard to E’’, there is a characteristic drop at the glass transition temperature (Tg). For recast Nafion, this drop is observed at around 120 °C, but the E″ drop is shifted to higher temperatures in the case of sSLM membranes, reflecting changes in the polymer’s segmental mobility due to filler incorporation. The superior thermal stability of nanocomposite membranes is further pointed out by the damping factor (tan δ = E″/E′) plots illustrated in Figure 8b. The peak of the tan δ curve is commonly used to determine the glass transition temperature (Tg) of the hydrophilic clusters of Nafion. The tan δ peak is observed at approximately 120–130 °C in the case of the filler-free membrane. The tan δ peaks for the sSLM-filled membranes are shifted to significantly higher temperatures, i.e., ca. 180 °C. This substantial upward shift in Tg with increasing filler loading indicates a significant restriction of polymer chain mobility, likely due to strong interfacial interactions between the Nafion polymer chains and the sSLM filler. A membrane that maintains its structural integrity and dimensional stability at elevated temperatures is less prone to physical degradation, which, combined with the optimized water management and proton conductivity, leads to superior and more durable fuel cell operation.

## 4. Conclusions

This study successfully demonstrated the synergistic enhancement of Nafion membranes through the incorporation of sulfonated silica layered materials (sSLMs), addressing critical challenges associated with high-temperature, low-humidity fuel cell operation. We systematically investigated the impact of sSLMs on the physico-chemical, electrochemical, and thermomechanical properties of the resulting nanocomposite membranes. The sSLM filler significantly improved the hydrophilic character of the Nafion matrix with a clear increase in both the IEC and water uptake capacity while simultaneously improving the dimensional stability. Water self-diffusion coefficient measurements consistently showed that the N-sSLM5 membrane maintained the highest self-diffusion coefficients across the entire temperature range up to 130 °C, confirming superior water retention. Proton conductivity tests revealed a marked superiority of sSLM-filled membranes under both fully humidified and dehydrating conditions. Crucially, under 120 °C and 20% RH, N-sSLM_5_ maintained significant conductivity at 30.24 mS cm^−1^, whereas pristine Nafion largely ceased to conduct. It was also observed that the incorporation of sSLM introduced a noticeable anisotropy in proton conductivity, with in-plane conductivity (σIP) being consistently higher than through-plane conductivity (σTP) for the nanocomposite membranes like N-sSLM_5_, in contrast to the isotropic behavior of filler-free Nafion. DMA highlighted the reinforcing effect of sSLM, with the glass transition temperature (Tg) shifting from approximately 120–130 °C for filler-free Nafion to circa 180 °C for sSLM-filled membranes. Ultimately, single-H_2_/O_2_ fuel cell performance validation underscored the practical benefits, as the N-sSLM_5_ membrane delivered a remarkable peak power density of approximately 340 mW cm^−2^ at 120 °C and 20% RH, representing a two-fold improvement compared to the 115 °C mW cm^−2^ achieved by the benchmark Nafion under the same challenging conditions. In summary, the strategic integration of sulfonated silica layered materials effectively mitigates the limitations of conventional Nafion membranes, leading to a new class of high-performance PEMs with enhanced proton conductivity, superior water management, and robust thermomechanical stability. These sSLM–Nafion nanocomposite membranes represent promising candidates for next-generation fuel cells, facilitating operation at higher temperatures and simplified humidification systems, thereby contributing to increased overall system efficiency and reduced cost. A crucial area for future work involves comprehensive investigations into their long-term durability and stability under realistic and accelerated fuel cell operating conditions. Such studies, including prolonged stress tests and in situ degradation analyses, are essential to fully ascertain their potential for commercial application and to optimize their lifetime performance.

## Data Availability

The original contributions presented in this study are included in the article. Further inquiries can be directed to the corresponding authors.

## References

[1] Mauritz K.A., Moore R.B. (2004). State of Understanding of Nafion. Chem. Rev..

[2] Karimi M.B., Mohammadi F., Hooshyari K. (2019). Recent Approaches to Improve Nafion Performance for Fuel Cell Applications: A Review. Int. J. Hydrogen Energy.

[3] Sengupta S., Lyulin A.V. (2019). Molecular Modeling of Structure and Dynamics of Nafion Protonation States. J. Phys. Chem. B.

[4] Privalov A.F., Sinitsyn V.V., Vogel M. (2023). Transport Mechanism in Nafion Revealed by Detailed Comparison of 1H and 17O Nuclear Magnetic Resonance Diffusion Coefficients. J. Phys. Chem. Lett..

[5] Sun H., Sun Z., Wu Y. (2012). Proton Transfer Mechanism in Perfluorinated Sulfonic Acid Polytetrafluoroethylene. Int. J. Hydrogen Energy.

[6] Peighambardoust S.J., Rowshanzamir S., Amjadi M. (2010). Review of the Proton Exchange Membranes for Fuel Cell Applications.

[7] Hooshyari K., Amini Horri B., Abdoli H., Fallah Vostakola M., Kakavand P., Salarizadeh P. (2021). A Review of Recent Developments and Advanced Applications of High-Temperature Polymer Electrolyte Membranes for Pem Fuel Cells. Energies.

[8] Ozen D.N., Timurkutluk B., Altinisik K. (2016). Effects of Operation Temperature and Reactant Gas Humidity Levels on Performance of PEM Fuel Cells. Renew. Sustain. Energy Rev..

[9] Borup R., Meyers J., Pivovar B., Kim Y.S., Mukundan R., Garland N., Myers D., Wilson M., Garzon F., Wood D. (2007). Scientific Aspects of Polymer Electrolyte Fuel Cell Durability and Degradation. Chem. Rev..

[10] Pham T.A., Nam L.V., Choi E., Lee M.S., Jun T.S., Jang S., Kim S.M. (2021). Mechanically Stable Thinned Membrane for a High-Performance Polymer Electrolyte Membrane Fuel Cell via a Plasma-Etching and Annealing Process. Energy Fuels.

[11] Dafalla A.M., Jiang F. (2018). Stresses and Their Impacts on Proton Exchange Membrane Fuel Cells: A Review. Int. J. Hydrogen Energy.

[12] Prykhodko Y., Fatyeyeva K., Hespel L., Marais S. (2021). Progress in Hybrid Composite Nafion®-Based Membranes for Proton Exchange Fuel Cell Application. Chem. Eng. J..

[13] Lu J.L., Fang Q.H., Li S.L., Jiang S.P. (2013). A Novel Phosphotungstic Acid Impregnated Meso-Nafion Multilayer Membrane for Proton Exchange Membrane Fuel Cells. J. Membr. Sci..

[14] Sizov V.E., Zefirov V.V., Abramchuk S.S., Korlyukov A.A., Kondratenko M.S., Vasil’ev V.G., Gallyamov M.O. (2020). Composite Nafion-Based Membranes with Nanosized Tungsten Oxides Prepared in Supercritical Carbon Dioxide. J. Membr. Sci..

[15] Krathumkhet N., Vongjitpimol K., Chuesutham T., Changkhamchom S., Phasuksom K., Sirivat A., Wattanakul K. (2018). Preparation of Sulfonated Zeolite ZSM-5/Sulfonated Polysulfone Composite Membranes as PEM for Direct Methanol Fuel Cell Application. Solid State Ion..

[16] Prapainainar P., Du Z., Theampetch A., Prapainainar C., Kongkachuichay P., Holmes S.M. (2020). Properties and DMFC Performance of Nafion/Mordenite Composite Membrane Fabricated by Solution-Casting Method with Different Solvent Ratio. Energy.

[17] Cui Y., Liu Y., Wu J., Zhang F., Baker A.P., Lavorgna M., Wu Q., Tang Q., Lu J., Xiao Z. (2018). Porous Silicon-Aluminium Oxide Particles Functionalized with Acid Moieties: An Innovative Filler for Enhanced Nafion-Based Membranes of Direct Methanol Fuel Cell. J. Power Sources.

[18] Truffier-Boutry D., De Geyer A., Guetaz L., Diat O., Gebel G. (2007). Structural Study of Zirconium Phosphate-Nafion Hybrid Membranes for High-Temperature Proton Exchange Membrane Fuel Cell Applications. Macromolecules.

[19] Donnadio A., Narducci R., Casciola M., Marmottini F., D’Amato R., Jazestani M., Chiniforoshan H., Costantino F. (2017). Mixed Membrane Matrices Based on Nafion/UiO-66/SO_3_H-UiO-66 Nano-MOFs: Revealing the Effect of Crystal Size, Sulfonation, and Filler Loading on the Mechanical and Conductivity Properties. ACS Appl. Mater. Interfaces.

[20] Zhang J., Bai H.J., Ren Q., Luo H.B., Ren X.M., Tian Z.F., Lu S. (2018). Extra Water- and Acid-Stable MOF-801 with High Proton Conductivity and Its Composite Membrane for Proton-Exchange Membrane. ACS Appl. Mater. Interfaces.

[21] Li J., Xu G., Luo X., Xiong J., Liu Z., Cai W. (2018). Effect of Nano-Size of Functionalized Silica on Overall Performance of Swelling-Filling Modified Nafion Membrane for Direct Methanol Fuel Cell Application. Appl. Energy.

[22] Oh K., Kwon O., Son B., Lee D.H., Shanmugam S. (2019). Nafion-Sulfonated Silica Composite Membrane for Proton Exchange Membrane Fuel Cells under Operating Low Humidity Condition. J. Membr. Sci..

[23] Matos B.R., Santiago E.I., Rey J.F.Q., Ferlauto A.S., Traversa E., Linardi M., Fonseca F.C. (2011). Nafion-Based Composite Electrolytes for Proton Exchange Membrane Fuel Cells Operating above 120 °C with Titania Nanoparticles and Nanotubes as Fillers. J. Power Sources.

[24] Jang S., Kang Y.S., Choi J., Yeon J.H., Seol C., Nam L.V., Choi M., Kim S.M., Yoo S.J. (2020). Prism Patterned TiO_2_ Layers/Nafion® Composite Membrane for Elevated Temperature/Low Relative Humidity Fuel Cell Operation. J. Ind. Eng. Chem..

[25] Burgaz E., Lian H., Alonso R.H., Estevez L., Kelarakis A., Giannelis E.P. (2009). Nafion–Clay Hybrids with a Network Structure. Polymer.

[26] Hasani-Sadrabadi M.M., Dashtimoghadam E., Majedi F.S., Kabiri K. (2009). Nafion®/Bio-Functionalized Montmorillonite Nanohybrids as Novel Polyelectrolyte Membranes for Direct Methanol Fuel Cells. J. Power Sources.

[27] Rhee C.H., Kim H.K., Chang H., Lee J.S. (2005). Nafion/Sulfonated Montmorillonite Composite: A New Concept Electrolyte Membrane for Direct Methanol Fuel Cells. Chem. Mater..

[28] Zhang W., Li M.K.S., Yue P.L., Gao P. (2008). Exfoliated Pt-Clay/Nafion Nanocomposite Membrane for Self-Humidifying Polymer Electrolyte Fuel Cells. Langmuir.

[29] Adjemian K.T., Dominey R., Krishnan L., Ota H., Majsztrik P., Zhang T., Mann J., Kirby B., Gatto L., Velo-Simpson M. (2006). Function and Characterization of Metal Oxide-Nafion Composite Membranes for Elevated-Temperature H 2/O 2 PEM Fuel Cells. Chem. Mater..

[30] Seo D.C., Jeon I., Jeong E.S., Jho J.Y. (2020). Mechanical Properties and Chemical Durability of Nafion/Sulfonated Graphene Oxide/Cerium Oxide Composite Membranes for Fuel-Cell Applications. Polymers.

[31] Berning T., Bessarabov D. (2023). GOMEA: A Conceptual Design of a Membrane Electrode Assembly for a Proton Exchange Membrane Electrolyzer. Membranes.

[32] Yin C., Xiong B., Liu Q., Li J., Qian L., Zhou Y., He C. (2019). Lateral-Aligned Sulfonated Carbon-Nanotubes/Nafion Composite Membranes with High Proton Conductivity and Improved Mechanical Properties. J. Membr. Sci..

[33] Nicotera I., Kosma V., Simari C., D’Urso C., Aricò A.S., Baglio V. (2015). Methanol and Proton Transport in Layered Double Hydroxide and Smectite Clay-Based Composites: Influence on the Electrochemical Behavior of Direct Methanol Fuel Cells at Intermediate Temperatures. J. Solid State Electrochem..

[34] Zhu L.Y., Li Y.C., Liu J., He J., Wang L.Y., Lei J.D. (2022). Recent Developments in High-Performance Nafion Membranes for Hydrogen Fuel Cells Applications. Pet. Sci..

[35] Enotiadis A., Boutsika L.G., Spyrou K., Simari C., Nicotera I. (2017). A Facile Approach to Fabricating Organosilica Layered Material with Sulfonic Groups as an Efficient Filler for Polymer Electrolyte Nanocomposites. New J. Chem..

[36] Nicotera I., Simari C., Boutsika L.G., Coppola L., Spyrou K., Enotiadis A. (2017). NMR Investigation on Nanocomposite Membranes Based on Organosilica Layered Materials Bearing Different Functional Groups for PEMFCs. Int. J. Hydrogen Energy.

[37] Simari C., Enotiadis A., Nicotera I. (2020). Transport Properties and Mechanical Features of Sulfonated Polyether Ether Ketone/Organosilica Layered Materials Nanocomposite Membranes for Fuel Cell Applications. Membranes.

[38] Cossari P., Simari C., Cannavale A., Gigli G., Nicotera I. (2018). Advanced Processing and Characterization of Nafion Electrolyte Films for Solid-State Electrochromic Devices Fabricated at Room Temperature on Single Substrate. Solid State Ion..

[39] Lufrano E., Simari C., Di Vona M.L., Nicotera I., Narducci R. (2021). How the Morphology of Nafion-Based Membranes Affects Proton Transport. Polymers.

[40] Springer T.E., Zawodzinski T.A., Gottesfeld S. (2008). Polymer Electrolyte Fuel Cell Model. J. Electrochem. Soc..

[41] Stejskal E.O., Tanner J.E. (1965). Spin Diffusion Measurements: Spin Echoes in the Presence of a Time-Dependent Field Gradient. J. Chem. Phys..

[42] Nicotera I., Kosma V., Simari C., Angioni S., Mustarelli P., Quartarone E. (2015). Ion Dynamics and Mechanical Properties of Sulfonated Polybenzimidazole Membranes for High-Temperature Proton Exchange Membrane Fuel Cells. J. Phys. Chem. C.

[43] Simari C., Lo Vecchio C., Baglio V., Nicotera I. (2020). Sulfonated Polyethersulfone/Polyetheretherketone Blend as High Performing and Cost-Effective Electrolyte Membrane for Direct Methanol Fuel Cells. Renew. Energy.

[44] Poiana R., Lufrano E., Tsurumaki A., Simari C., Nicotera I., Navarra M.A. (2021). Safe Gel Polymer Electrolytes for High Voltage Li-Batteries. Electrochim. Acta.

[45] Simari C., Vecchio C.L., Enotiadis A., Davoli M., Baglio V., Nicotera I. (2019). Toward Optimization of a Robust Low-Cost Sulfonated-Polyethersulfone Containing Layered Double Hydroxide for PEM Fuel Cells. J. Appl. Polym. Sci..

[46] Simari C., Enotiadis A., Lo Vecchio C., Baglio V., Coppola L., Nicotera I. (2020). Advances in Hybrid Composite Membranes Engineering for High-Performance Direct Methanol Fuel Cells by Alignment of 2D Nanostructures and a Dual-Layer Approach. J. Membr. Sci..

